# Authors' reply to letter regarding “Inflammaging links life's essential 8 to white matter brain aging”

**DOI:** 10.1016/j.ebiom.2025.105909

**Published:** 2025-09-13

**Authors:** Tianzhou Ma, Zhenyao Ye, Li Feng, Shuo Chen

Dear Editors,

We want to thank Dr. Grimolizzi's letter following up our study on Life's Essential 8 (LE8) and white matter brain aging.^1^ It is indeed of clinical interest to learn the underlying biological mechanism on how higher LE8 score helps maintain the integrity of white matter. We have previously found allostatic load (AL), a multi-system measure of the cumulative “wear and tear” on the body which includes inflammation,^2^ accelerated white matter brain aging.^3^

We concur with the suggestion to hypothesize inflammaging as a potential mediation pathway that explains the effect of LE8 on brain aging. We retrieved the data of three inflammaging biomarkers, C-reactive protein (CRP), glycoprotein acetyls and white blood cell (WBC) count measured at baseline from UK Biobank (UKB). We tested on the pathway: LE8-> inflammaging ->white matter brain aging via causal mediation analysis using the R package “mediation”.^4^ Among them, we found CRP accounted for ∼8% of the total effect of LE8 on white matter brain aging (p = 0.004), WBC count accounted for ∼ 7% (p = 0.004), while glycoprotein acetyls was not found as a significant mediator. We further found higher LE8 tends to lower the inflammaging level which in turns helps protect our white matter integrity.

The findings provided partial support for the hypothesized pathway though the mediation effects by the available inflammaging biomarkers were relatively weak. The cross-sectional nature of the data makes it challenging to infer the causal mediating role of inflammaging. Future research should investigate the pathway by tracking the lifestyle, inflammaging and white matter change over time, and evaluate a broader spectrum of biomarkers characterizing inflammaging to confirm the hypothesis.

## Contributors

T.M. and S.C. have conceputalized the idea. T.M. has taken the lead in drafting the letter. Z.Y. and L.F. have performed the analysis. All authors have contributed to the final editing of the letter.

## Declaration of interests

None.
